# *Garcinia kola* Nuts: A Molecular Cocktail for Skin Care

**DOI:** 10.3390/molecules30183813

**Published:** 2025-09-19

**Authors:** Durand Dah-Nouvlessounon, Coline Fernandes, Ronald Alonso Salas Araya, Lamine Baba-Moussa, Rodica Mihaela Dinica, Ahcène Boumendjel

**Affiliations:** 1 Laboratory of Biology and Molecular Typing in Microbiology, Department of Biochemistry and Cell Biology, Faculty of Sciences and Techniques, University of Abomey-Calavi, Cotonou 05BP1604, Benin; dahdurand@gmail.com (D.D.-N.); laminesaid@yahoo.fr (L.B.-M.); 2 Univ. Grenoble Alpes, INSERM, LRB, 38000 Grenoble, France; coline.fernandes@univ-grenoble-alpes.fr (C.F.); ronald-alonso.salas-araya@univ-grenoble-alpes.fr (R.A.S.A.); 3 Department of Chemistry, Physics and Environment, “Dunarea de Jos” University of Galati, 47 Domneasca Street, 800008 Galati, Romania

**Keywords:** *G. kola*, nuts, dermocosmetics, unsaturated fatty acids, garcinoic acid, biflavanones

## Abstract

*Garcinia kola* is an emblematic tree used in traditional medicine in many regions in Africa. In particular, its nuts are commonly used for the management of various diseases. However, to the best of our knowledge, *G. kola* nuts have never been investigated as potential sources of active ingredients in dermocosmetics. In this paper, nuts from *G. kola* were investigated to shed light on the extraction, purification and characterization of three components with potential dermocosmetic applications. The nuts were subjected to extraction with different solvents, including cyclohexane, dichloromethane, ethyl acetate, and methanol. Each extract was purified by column chromatography on silica gel. Pure compounds were characterized by NMR and mass spectrometry and comparison with reported literature data. Unsaturated fatty acids were found in the cyclohexane and dichloromethane extract, garcinoic acid (a vitamin E derivative) in the dichloromethane extract, and the biflavanone GB1 in the methanol extract. The presence of unsaturated fatty acids, garcinoic acid, and biflavanone in the nuts of *G. kola* as dominant compounds suggests that this plant material holds potential to be used for the development of active compounds for skin care and well-being.

## 1. Introduction

**Figure 1:**
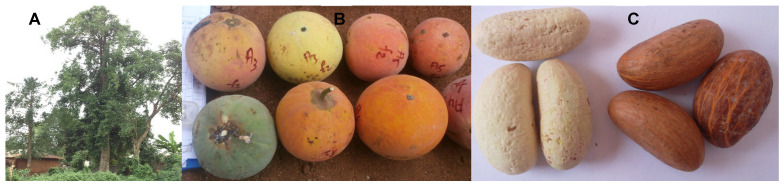
*Garcinia kola*. Tree (**A**), fruits (**B**), and nuts (**C**).

*Garcinia kola* (Clusiaceae) is a widely distributed tree in many tropical regions throughout West and Central Africa [1], where it is commonly known as bitter kola. Beyond its ethnobotanical relevance, *G. kola* holds a special place in the social life of local populations. Nuts are traditionally offered at wedding ceremonies or as tokens of respect and appreciation. In addition to its societal uses, many parts of *G. kola* are used in folk medicine to treat diverse diseases [1]. The fruit is a drupe of 5–10 cm in diameter and weighs 30–50 g, Figure 1 [2]. It is usually smooth and contains a yellow-red pulp. Each fruit may contain up to five nuts. These nuts are highly valued and frequently chewed by local populations, mostly due to their caffeine content [3]. The US Food and Drug Administration (FDA) has classified *G. kola* nuts as safe for human consumption and has approved kola extract as a flavor agent in pharmaceutical preparations [4].

Pharmacological investigation of extracts from *G. kola* nuts has demonstrated a broad range of biological activities, including antitrypanosomal effects [5], anti-inflammatory activity [6], neuroprotective effect [7], antiplasmodial activity [8], and antiulcer properties [9]. Interestingly, potent antibacterial and cytotoxic cytochalasins were isolated from endophytic fungi harbored in *G. kola* nuts [10]. At the chemical level, several phytochemical investigations were conducted on *G. kola* which revealed the presence of a wide variety of secondary metabolites, including unsaturated fatty acids, benzophenones, benzopyrans, polyphenols, vitamin E derivatives, and sterols [11]. Hence, owing to their traditional use and the reported biological activities, *G. kola* nuts are of interest to researchers.

As part of our work aimed at promoting the flora of Benin, we were interested in Garcinia kola nuts and the possibility of finding methods for economic valuation. Our attention has focused on the possibility of using products derived from *G. kola* for dermocosmetic use. Our interest in the potential use of *G. kola* nuts in cosmetics was motivated by the presence of a group of secondary metabolites known for their positive effect on skin, as reported in the literature [11]. Among the compounds of interest reported in *Garcinia kola* are unsaturated fatty acids such as oleic and linoleic acid, vitamin E derivatives such as garcinoic acid, and polyphenols such as the biflavanone GB1.

The objective of this work is to carry out a phytochemical investigation of extracts of *Garcinia kola* nuts to check for the concomitant presence of oleic and linoleic acids, garcinoic acid, and biflavanone GB1 with the aim of considering these extracts in the context of multifunctional cosmetics.

## 2. Results and Discussion

### 2.1. Extraction

The dried and ground powder of *G. kola* nuts was subjected to successive extraction with four organic solvents of increased polarities: cyclohexane, dichloromethane, ethyl acetate, and methanol (see Section 4). The extractions were performed under identical conditions regarding temperature, extraction time, and solvent volume. The yield of each extraction is summarized in Table 1. Each extract was submitted to a TLC analysis to check for the presence of possible dominant compounds.

### 2.2. Isolation of Fatty Acids

**Figure 2:**
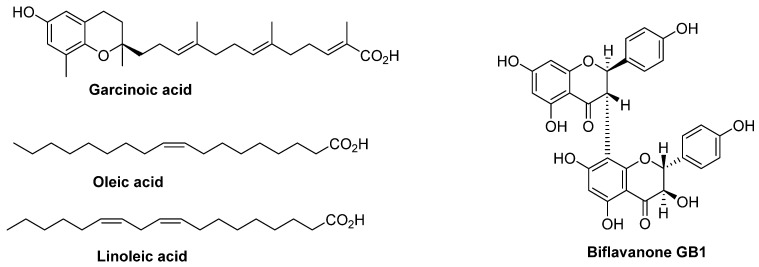
Structures of isolated compounds from the nuts of *G. kola*.

The cyclohexane extract was subjected to chromatographic purification on silica gel using a solvent gradient (cyclohexane–dichloromethane), providing a mixture of two compounds (135 mg). The ^1^H NMR analysis showed the presence of unsaturated fatty acids, evidenced by the presence of characteristic signals corresponding to olefinic protons at around 5.5 ppm and a high number of aliphatic protons at upfield chemical shift [12]. Previous studies conducted on garcinia kola showed the presence of oleic and linoleic acids [13]. This guided us to compare the chromatographic profile (TLC) of isolated compounds to commercial oleic and linoleic acids, and we concluded that the two fatty acids isolated from the cyclohexane extract are oleic acid and linoleic acid (Figure 2). Since the mentioned fatty acids have already been isolated from garcinia, we did not consider it useful to further investigate the structural study of isolated fatty acids. It should be highlighted that oleic and linoleic acids were also present in the dichloromethane extract according to TLC analysis.

### 2.3. Isolation of Garcinoic Acid

The purification of the dichloromethane extract revealed the presence of one main compound which was isolated in pure form by chromatography purification (78 mg). The mass spectrometry performed by negative ion electrospray (ESI^−^) displayed a main peak at 425.8 corresponding to [M-H]^+^. The ^1^H NMR spectrum exhibited four patterns of protons: three signals in the aromatic region, two allylic protons around 5.1 ppm characteristic of a -C=C**H**-CH_2_-C system, five methyl groups, and aliphatic protons. Among the signals in the aromatic region, there is one triplet corresponding to a coupling of a proton with two adjacent ones. The two remaining signals in the aromatic regions are two doublets with coupling constants characteristic (*J* = 2–3 Hz) of protons at meta position relative to a phenyl ring.

Among the methyl groups, we distinguish one methyl displaying a chemical shift at 2 ppm, characteristic of a CH_3_ linked to an aromatic ring. The remaining signals correspond to 14 protons linked to saturated carbons. The ^1^H NMR data are clearly in favor of a polyunsaturated long chain combined with a substituted phenyl ring. The ^13^C NMR spectrum showed the presence of allylic carbons, methyl groups, a carboxylic acid carbon, aromatic carbons bearing oxygens, and saturated carbons. Taken together, the ^13^C NMR data was consistent with what was deduced from the ^1^H NMR. Without further 2D NMR investigation, the structure of garcinoic acid was considered, as this compound was previously isolated from *Garcinia kola* nuts. Hence, the structure of garcinoic acid was confirmed by comparing our NMR data with the literature [14] (Figure 2). It should be highlighted that garcinoic acid was also detected in the ethyl acetate extract but at a much lower level than in the dichloromethane extract.

### 2.4. Isolation of Biflavanone GB1

The methanolic extract was subjected to purification on silica gel chromatography. One main compound (369 mg) was isolated in pure form as brown powder, for which the ^1^H NMR recorded in DMSO-*d*_6_ showed the presence of a high number of aromatic and phenolic protons, which are in full agreement with a polyphenol scaffold. The ^1^H NMR spectra recorded at room temperature in CD_3_OD showed seven exchangeable protons: two at low field (around 12 ppm), two in the range of 10.5 to 11.5 ppm, two around 9.5 ppm, and one at 5.8 ppm. The presence of phenol signals at higher chemical shifts is a hallmark of hydroxy groups at position 5 of a flavone or flavanone scaffold. A dimeric structure is deduced due to the splitting of all signals. At room temperature, the NMR spectrum exhibited signal doubling due to the coexistence of two conformers, complicating direct interpretation.

The structure of a biflavanone was considered because it was already reported and fully identified in Garcinia kola nuts. The dimer bridge 3→8″ was identified based on the ^13^C chemical shifts of carbons C-2, C-3 and C-8 compared to those of the monomer, naringenin. This was elegantly demonstrated by Kumar et al. [15]. The structure was fully identified through further ^13^C NMR and 2D NMR (HSQC, HMBC) analyses and was in full agreement with the biflavanone GB1 shown in Figure 2 [16].

Based on the quantities of the three pure compounds relative to the initial plant material used during the extraction (72 g), the yields of compounds are as follows: 0.18, 0.10, and 0.50% for oleic and linoleic acids, garcinoic acid, and GB1, respectively.

Our results confirmed the dominant and concomitant existence of unsaturated fatty acids; garcinoic acids, namely d-tocotrienoloic acid; and the biflavanone GB1 in *Garcinia kola* nuts. Although these substances were previously isolated from different parts of *Garcinia kola* according to different extraction procedures, this is the first report to describe their concomitant presence in *Garcinia kola* nuts extracts. Using extracts from *Garcinia kola* nuts in dermocosmetics can be considered in the context of multifunctional cosmetics as a growing strategy [17].

The literature demonstrating the interest of the three compounds for cosmetics is very rich. Garcinoic acid is a vitamin E analog, structurally very close to d-tocotrienol and a-tocopherol 13′-carboxylic acid. Vitamin E and its derivatives have emerged as active compounds for the treatment of several skin disorders due to their antioxidant properties, accounting for many skin effects including photoprotection, skin aging reduction, pyrimidine dimers reduction, and DNA repair [18,19,20,21]. The effect of vitamin E derivatives on enhancing collagen synthesis and inhibiting collagen degradation was reported [22,23]. Moreover, the effect of tocotrienol on pigmentation, moisture, wrinkles, and UV exposure was described [24,25].

Flavanones are well known as actives in skin care products, mainly due to their antioxidant activity [26,27,28], inflammatory effects [29,30], skin elasticity through elastase inhibition [31], and anti-collagenase activity [32].

Unsaturated fatty acids are considered as allied compounds in dermocosmetics [33]. Studies based on animal or skin cell models suggest that unsaturated fatty acids such as linoleic acid, topically applied, repair the skin barrier, contribute to wound healing, and have photoprotective and anti-inflammatory effects [34]. Moreover, unsaturated fatty acids are frequently incorporated into lipid-based formulations in cosmetic products [35].

## 3. Conclusions

The main objective of this work was to provide evidence of the potential exploration of *G. kola* nuts in the development of skin care products through the presence of three known compounds: unsaturated fatty acids, biflavanone, and garcinoic. We showed that the three types of compounds are present in *Garcinia kola* nuts. However, it remains to be demonstrated whether the three types of compounds can be co-extracted without using undesirable solvents for cosmetic products. In this regard, ethanol could be a valuable choice. Supercritical CO_2_ can be an effective solvent, and is currently used in various applications, such as the decaffeination of coffee beans, the production of essential oils, and the manufacture of medicines. Moreover, ultrasound-assisted extraction could be applied. The latter has emerged as a simple and highly reproducible technique capable of extracting phytochemicals. Currently, we are exploring the use of the latter method to shed light on new components that constitute valuable ingredients.

## 4. Experimental Section

### 4.1. Materials and Methods

The fruits of *Garcinia kola* were collected in the commune of Adjarra in the district of Malanhoui in the South of Benin, more precisely between the coordinates 06°29′30.1″ N and 002°40′04.3″ E. These specimens were then deposited for official certification at the National Herbarium of Benin, located at the University of Abomey-Calavi in Cotonou, under the supervision of the curator of the Herbarium, Prof. Hounnankpon Yedomonhan. The fruits were then cut to separate the nuts from the pulp. These nuts were peeled and cut into small pieces before being subjected to drying under air conditioning at 20 ± 2 °C for 14 days. After drying, these samples were finely ground using a Retsch mill (model SM 2000/1430/Upm/Smf, Haan, Germany) to facilitate extraction. HPLC-grade solvents used for the extraction and purifications were purchased from Carl Roth and were used directly without further treatment. Purification was performed using a PuriFLASH 4250 system (Advion Interchim Scientific, Montluçon, France). NMR was recorded on a 400 MHz Bruker Avance-400 instrument (Mannheim, Germany). NMR experiments were run at 25 °C. Chemical shifts (*δ*) are reported in parts per million (ppm) relative to TMS as internal standard or relative to the solvent [^1^H: *δ*(CDCl_3_) = 7.26 ppm, *δ*(CD_3_OD) = 3.31 ppm *δ*(DMSO-*d*_6_) = 2.50 ppm. Mass spectra were performed by using either electrospray ionization (ESI) mass spectra on an Q Extractive Plus Orbitrap, Thermo Fisher scientific (or APCI, Advion Interchim Scientific, Montluçon, France). 

### 4.2. Preparation of Extracts

The maceration method using an increasing polarity gradient of four solvents (cyclohexane, dichloromethane, ethyl acetate, and methanol) was used. For the extraction process, 72 g of powder was mixed under continuous stirring with 300 mL of cyclohexane at room temperature. After 4 h of stirring at room temperature, the temperature was raised to 45 °C under a condenser. After 20 h of stirring at 45 °C, the mixture was filtered with filter paper (Whatman, Grade 114, 25 µm). The filtrate was concentrated using a rotary evaporator. The product obtained was dried under a vacuum and constitutes the crude hexane extract. The marc recovered was treated with CH_2_Cl_2_ (300 mL) in the same conditions as with cyclohexane. The process was repeated with ethyl acetate (residue recovered; treated with 300 mL of solvent for 4 h at room temperature then for 20 h at 45 °C). Finally, the experiment was repeated with methanol (300 mL). We performed one extraction cycle with each solvent. The yield of the products obtained was calculated by the ratio between the mass of the crude extract obtained and that of the powder of *G. kola* nuts.

### 4.3. Purification

Purification was performed by prep-HPLC using a PuriFLASH 4250 system (Interchim) equipped with a silica gel and a UV detector set at 254 nm. The silica gel column characteristics were a particle size of 30 mm and a pore diameter of 60 Å. Prior to purification, preliminary optimization to ensure efficient separation and purity was conducted in order to identify the most performant solvent. Hence, we tested different solvent systems using a combination of two of the following solvents: cyclohexane, ethyl acetate, dichloromethane, and methanol. For the medium-polarity extracts (cyclohexane and ethyl acetate), we used a gradient elution starting with a 1:1 mixture to initiate separation, followed by a gradual increase. The method was as follows: Time 0–3 min: Hold at 50% cyclohexane/50% ethyl acetate. Time 3–5 min: Ramp linearly from 50% to 75% ethyl acetate. Time 5–10 min: Ramp linearly from 75% to 90% ethyl acetate. Time 10–24 min: Hold at 90% ethyl acetate/10% cyclohexane to ensure all polar compounds are eluted. For the polar methanol extract, an isocratic method (constant solvent strength) was sufficient for separation. The method was held constant at 90% dichloromethane/10% methanol throughout the run

### 4.4. Structural Characterization

(2E,6E,10E)-13-[(2R)-6-hydroxy-2,8-dimethyl-3,4-dihydrochromen-2-yl]-2,6,10-trimethyltrideca-2,6,10-trienoic acid (Garcinoic acid)

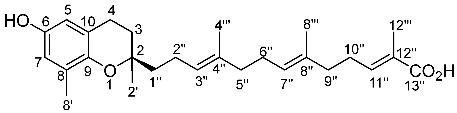



^1^H NMR (400 MHz, CD_3_OD): registered at room temperature. δ = 1.25 (3H, s, CH_3_-2′), 1.57 (3H, s, CH_3_-4‴), 1.49–1.56 (2H, m, CH_2_-1″), 1.59 (3H, s, CH_3_-8‴), 1.71–1.78 (2H, m, CH_2_-3), 1.80 (3H, s, CH_3_-12‴), 1.99–2.02 (2H, m, CH_2_-5″), 2.01 (3H, s, CH_3_-8′), 2.07–2.15 (6H, m, CH2-2″, CH_2_-6″, CH_2_-9″), 2.29 (2H, q, *J* = 7.2 Hz, CH_2_-10″), 2.70 (2H, t, *J* = 7.2 Hz, CH_2_-4), 5.12–5.17 (2H, m, CH-3″, CH-7″), 6.32 (1H, d, *J* = 2.7 Hz, H-5), 6.41 (1H, d, *J* = 2.68 Hz, H-7), 6.76 (1H, dt, *J*_1_ = 1.5 Hz, *J*_2_ = 7.1 Hz, H-11″). ^13^C NMR (100 MHz, CD_3_OD): δ = 12.3 (C-12‴), 15.7 (C-4‴), 15.9 (C-8‴), 16.2 (C-8′), 22.3 (C-2″), 22.2 (C-4), 24.4 (C-2′), 26.4 (C-6″), 27.4 (C-10″), 31.0 (C-3), 38.0 (C-9″), 39.4 (C-5″), 39.4 (C-1″), 75.3 (C-2), 112.3 (C-5), 115.2 (C-7), 121.7 (C-10), 124.6 (C-3″), 125.2 (C-7″), 126.7 (C-12″), 127.7 (C-8), 133.7 (C-8″), 134.9 (C-4″), 144.8 (C-11″), 146.0 (C-9), 148.1 (C-6), 172.3 (C-13″). LRMS (ESI-) *m*/*z* (%) 425 (100) [M-H]^+^, 172 (55).

3″,4′,4‴,5,5″,7,7″-heptahydroxy-3,8″-biflavanone (Biflavanone GB1)

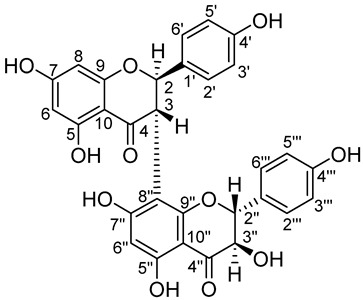



^1^H NMR (400 MHz, DMSO-*d*_6_): registered at room temperature. δ = 3.98 (0.5H, d, *J* = 10.4 Hz, CH-3″), 4.21 (0.5H, d, *J* = 10.4 Hz, CH-3″), 4.40 (0.5H, d, *J* = 11.92 Hz, CH-3), 4.63 (0.5H, d, *J* = 11.96 Hz, CH-3), 4.95 (0.5H, d, *J* = 11.4 Hz, CH-2″), 5.12 (0.5H, d, *J* = 11.12 Hz, CH-2″), 5.29 (0.5H, d, *J* = 11.88 Hz, CH-2), 5.63 (0.5H, d, *J* = 11.84 Hz, CH-2), 5.72–5.95 (4H, m, CH-6, CH-8, CH-6″, 3″-OH), 6.64 (2H, d, *J* = 7.2 Hz, CH-3‴, CH-5‴), 6.74 (1H, d, *J* = 7.5 Hz, CH-3′, CH-5′), 6.83 (1H, d, *J* = 7.5 Hz, CH-3′, CH-5′), 7.09 (2H, d, *J* = 7.3 Hz, CH-2‴, CH-6‴), 7.16 (2H, d, *J* = 7.3 Hz, CH-2′, CH-6′), 9.44 (0.5H, s, C_4′_-OH/C_4‴_-OH), 9.47 (0.5H, s, C_4′_-OH/C_4‴_-OH), 9.56 (0.5H, s, C_4′_-OH/C_4‴_-OH), 9.58 (0.5H, s, C_4′_-OH/C_4‴_-OH), 10.73–11.4 (2H, 4xls, C_7_-OH/C_7″_-OH), 11.73 (0.5H, s, C_5_-OH/C_5″_-OH), 11.85 (0.5H, s, C_5_-OH/C_5″_-OH), 12.16 (0.5H, s, C_5_-OH/C_5″_-OH), 12.29 (0.5H, s, C_5_-OH/C_5″_-OH). ^13^C NMR (100 MHz, DMSO-*d_6_*): δ = 47.3 (C-3), 72.3 (C-3″), 72.8 (C-3″), 81.6 (C-2), 82.1 (C-2), 82.9 (C-2″), 95.3 (C-6″), 95.4 (C-6″), 95.7 (C-8), 96.2 (C-6), 96.5 (C-6), 100.1 (C-10/C-10″), 100.6 (C-10/C-10″), 101.5 (C-8″), 101.7 (C-8″), 115.0 (C-3′/C-5′, C-3‴/C-5‴), 115.1 (C-3′/C-5′, C-3‴/C-5‴), 127.9 (C-1‴), 128.2 (C-1‴), 128.2 (C-1′), 128.3 (C-1′), 128.6 (C2′/C-6′), 129.1 (C2′/C-6′), 129.3 (C-2‴/C-6‴), 129.5 (C-2‴/C-6‴), 157.7 (C-4′/C-4‴), 157.9 (C-4′/C-4‴), 158.1 (C-9/C-9″), 158.2 (C-9/C-9″), 159.8 (C-9/C-9″), 160.6 (C-9/C-9″), 161.7 (C-5/C-5″), 163.2 (C-5/C-5″), 163.3 (C-5/C-5″), 164.4 (C-5/C-5″), 165.0 (C-7/C-7″), 165.5 (C-7/C-7″), 166.7 (C-7/C-7″), 166.8 (C-7/C-7″), 196.8 (C-4/C-4″), 197.1 (C-4/C-4″), 197.9 (C-4/C-4″). LRMS (APCI) *m*/*z* (%) 559.7 (5) [M-H]^+^, 423.7 (15), 271.4 (13), 153.2 (100%).

## Figures and Tables

**Table 1:** Yield of crude extracts from *G. kola* nuts (72 g).

Solvent	Extract Quantity (g)	Yield (%)
Cyclohexane	1.272	1.76
Dichloromethane	0.582	0.80
Ethyl acetate	2.993	4.15
Methanol	7.081	9.83

## Data Availability

Data are contained within the article and Supplementary Materials.

## Supplementary Materials

The following supporting information can be downloaded at https://www.mdpi.com/article/10.3390/molecules30183813/s1, File S1. NMR spectra of garcinoic acid, NMR spectra of GB1.
