# Supplementary material for: Garcinia kola Nuts: A Molecular Cocktail for Skin Care
Source: Molecules. 2025 Sep 19;30(18):3813. doi: 10.3390/molecules30183813 (PMC12472956; doi:10.3390/molecules30183813)

Garcinia kola Nuts: A Molecular Cocktail for Skin Care

Durand Dah-Nouvlessounon [1], Coline Fernandes [2,†], Ronald Alonso Salas Araya [2,†], Lamine Baba-Moussa [1], Rodica Mihaela Dinica [3] and Ahcène Boumendjel [2,*]

Characterization of pure compound isolated from the dichloromethane extract (Garcinoic acid)

MS (Atmospheric pressure chemical ionization)

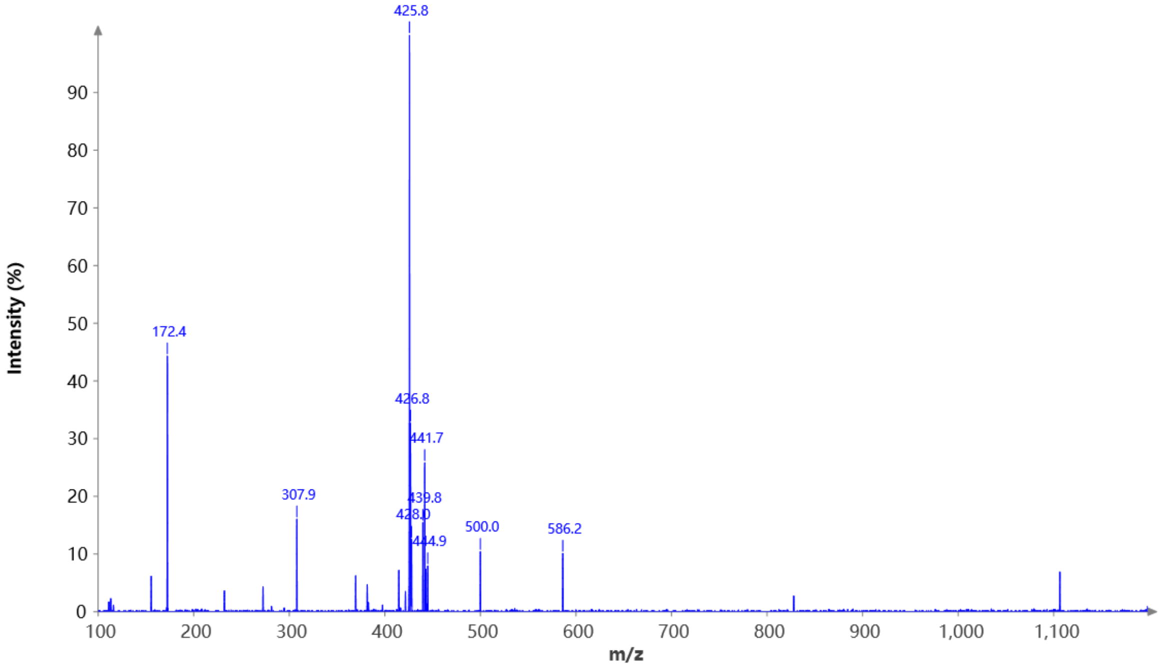

^{1}H NMR

Recorded in DMSO-d_6 (400 MHz, room temperature, ~ 10 mg/mL)

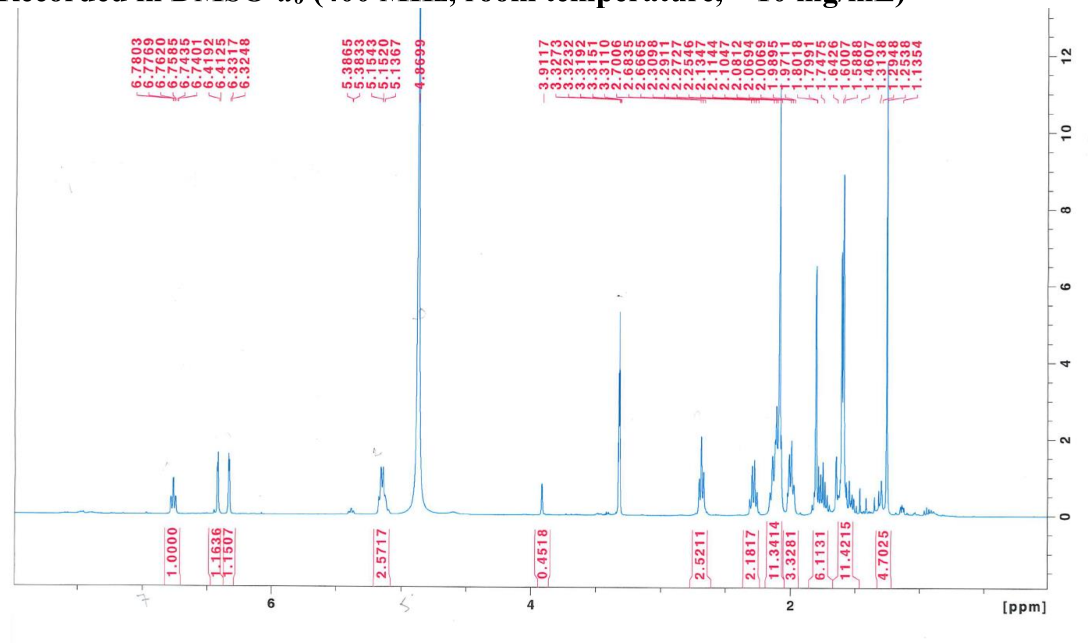

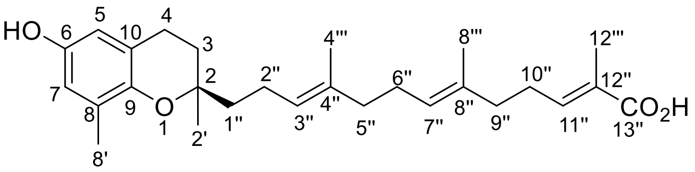

	^{1}H	^{13}C
**1**	-	-
**2**	-	75.3
**2’**	1.25 (s)	24.4
**3**	1.71-1.78 (m)	31.0
**4**	2.70 (t, J = 7.2 Hz)	22.2
**5**	6.32 (1H, d, J = 2.7 Hz)	112.3
**6**	-	148.1
**7**	6.41 (1H, d, J = 2.68 Hz)	115.2
**8**	-	127.7
**8’**	2.01 (s)	16.2
**9**	-	146.0
**10**	-	121.7
**1”**	1.49-1.56 (m)	39.4
**2”**	2.07-2.15 (m)	22.3
**3”**	5.12-5.17 (m)	124.6
**4”**	-	134.9
**4”’**	1.57 (s)	15.7
**5”**	1.99-2.02 (m)	39.4
**6”**	2.07-2.15 (m)	26.4
**7”**	5.12-5.17 (m)	125.2
**8”**	-	133.7
**8”’**	1.59 (s)	15.9
**9”**	2.07-2.15 (m)	38.0

**10”**	2.29 (q, J = 7.2 Hz)	27.4
**11”**	6.76 (dt, J_1 = *1.5 Hz*, J_2 = *7.1* Hz)	144.8
**12”**	**-**	126.7
**12”’**	1.80 (s)	12.3
**13**	**-**	172.3

Characterization of pure compound isolated from the methanol extract (Biflavanone GB1)

MS (Atmospheric pressure chemical ionization)

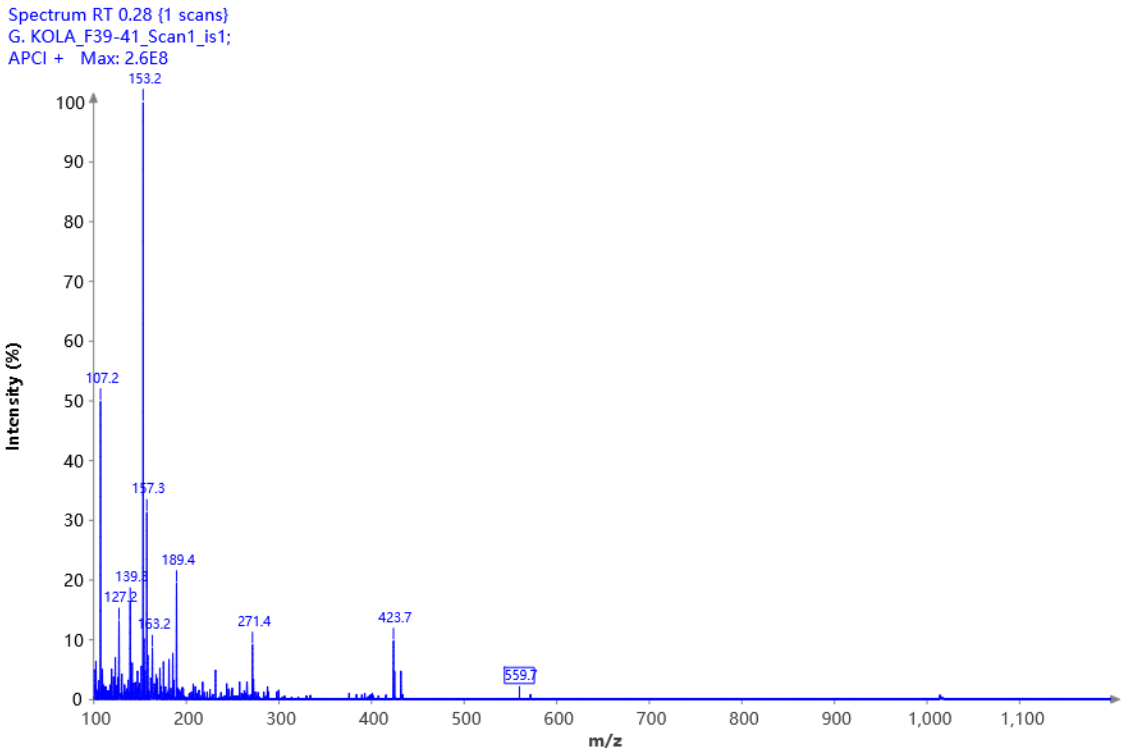

NMR Assignments

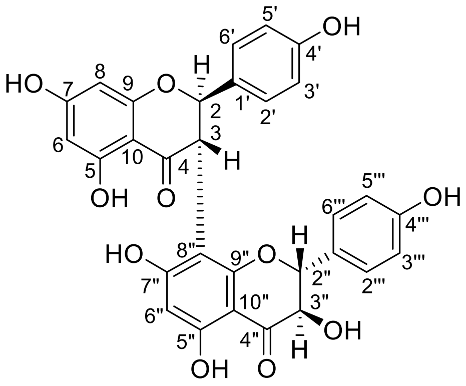

	^{1}H<br>^{1}H NMR Recorded in DMSO-d_6 (400 MHz, room temperature, ~ 10 mg/mL)	^{13}C<br>Recorded in DMSO-d_6 (100 MHz, room temperature, ~ 10 mg/mL)

1	-	
2	5.29 (0.5H, d, J = 11.88 Hz)<br>5.63 (0.5H, d, J = 11.84 Hz)	81.6<br>82.1
3	4.40 (0.5 H, d, J = 11.92 Hz)<br>4.63 (0.5H, d, J = 11.96 Hz)	47.3
4	-	196.8<br>197.1
5	-	161.7<br>163.3
6	5.72-5.95	96.2<br>96.5
7	-	165.0<br>166.5
8	5.72-5.95	95.7<br>96.2
9	-	158.1<br>158.2
10	-	100.1<br>100.6
1’	-	128.2<br>128.3
2’	7.16 (d, J = 7.3 Hz)	128.6<br>129.1
3’	6.74 (d, J = 7.5 Hz)	115.0<br>115.1
4’	-	157.7<br>157.9
5’	6.74 (d, J = 7.5 Hz)	115.0<br>115.1
6’	7.16 (d, J = 7.3 Hz)	128.6<br>129.1
1”	-	
2”	4.95 (0.5 H, d, J = 11.4 Hz)	82.9

	5.12 (0.5H, d, *J* = 11.12 Hz)	
3''	3.98 (0.5 H, d, *J* = 10.4 Hz)<br>4.21 (0.5 H, d, *J* = 10.4 Hz)	72.3<br>72.8
4''	-	196.8<br>197.1
5''	-	161.7<br>163.3
6''	5.72-5.95	95.3<br>95.4
7''	-	165.0<br>166.5
8''	-	101.5<br>101.7
9''	-	158.1<br>158.2
10''	-	100.1<br>100.6
1'''	-	127.9<br>128.1
2'''	7.09 (d, *J* = 7.3 Hz)	129.3<br>129.5
3'''	6.64 (d, *J* = 7.2 Hz)	115.0<br>115.1
4'''	-	157.7<br>157.9
5'''	6.64 (d, *J* = 7.2 Hz)	115.0<br>115.1
6'''	7.09 (d, *J* = 7.3 Hz)	129.3<br>129.5

^{1}H NMR Recorded in DMSO-d_6 (400 MHz, room temperature, ~ 10 mg/mL)

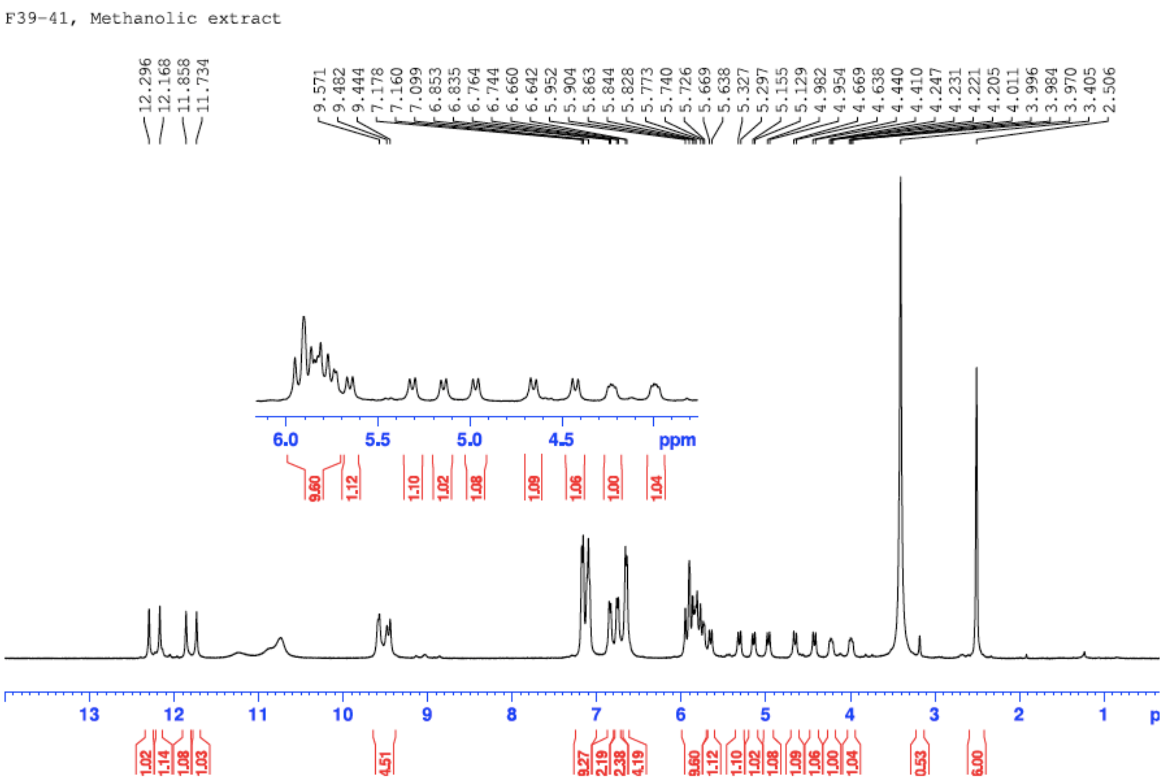

^{13}C NMR Recorded in DMSO-d_6 (100 MHz, room temperature, ~ 10 mg/mL)

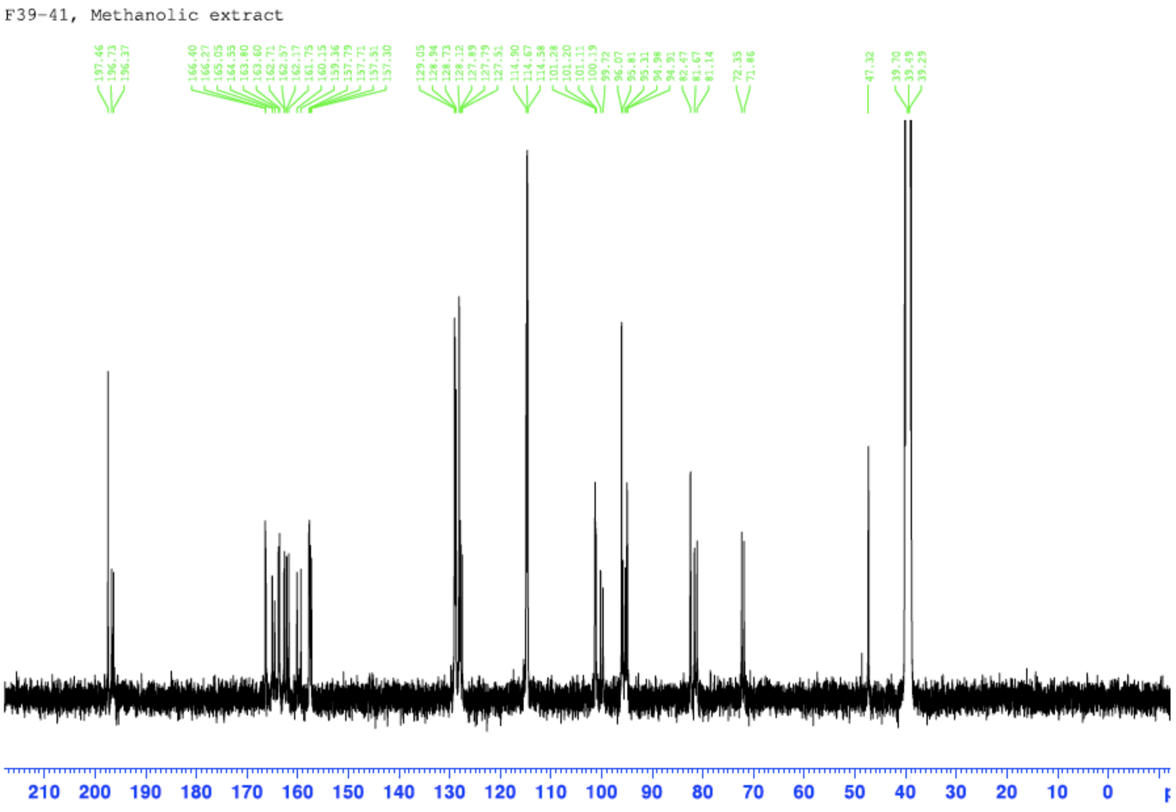

HSQC: Recorded in DMSO-d_6 (room temperature, ~ 10 mg/mL)

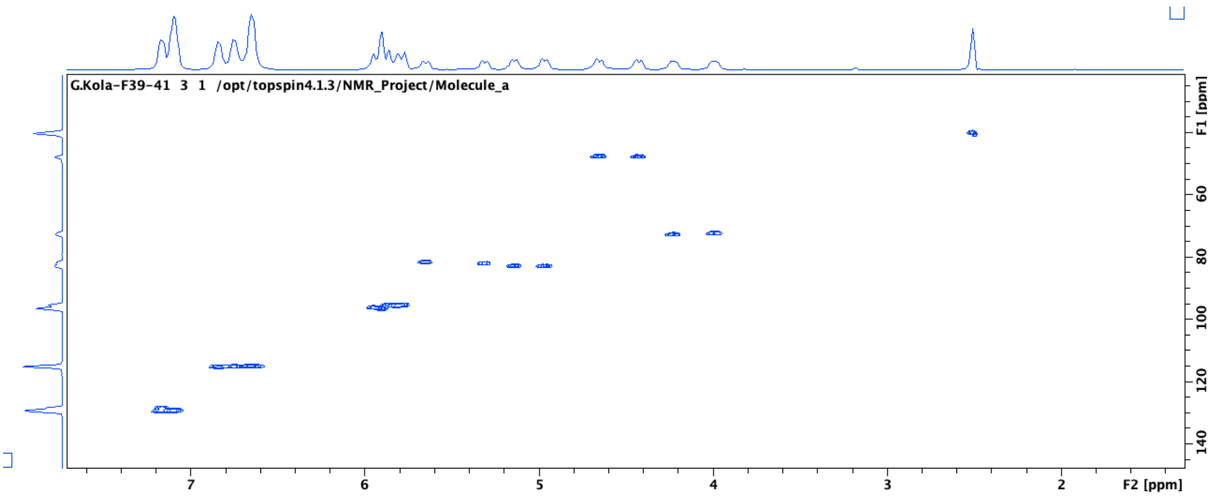

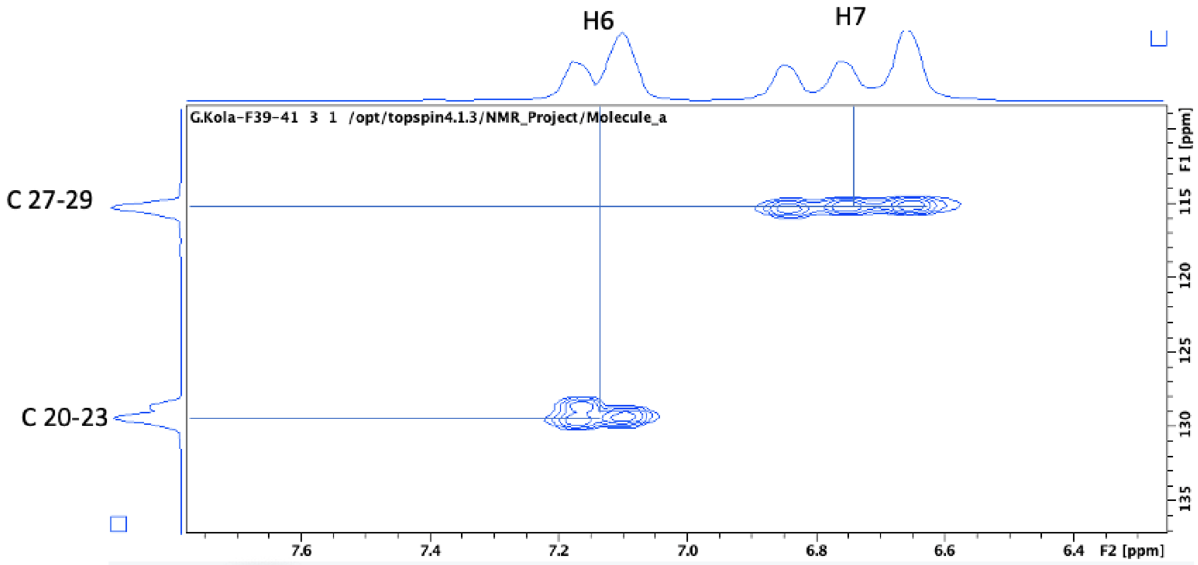

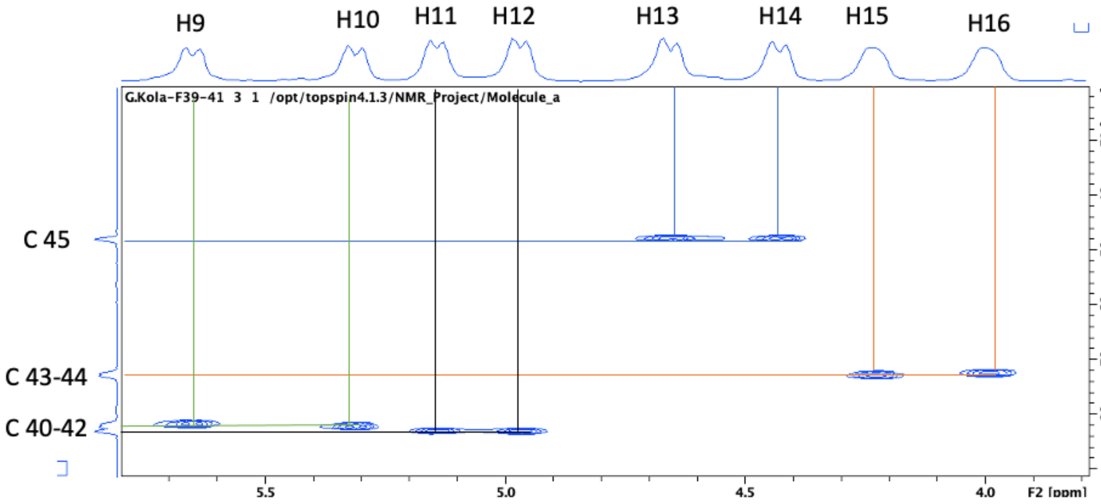

HMBC: Recorded in DMSO-d_6 (room temperature, ~ 10 mg/mL)

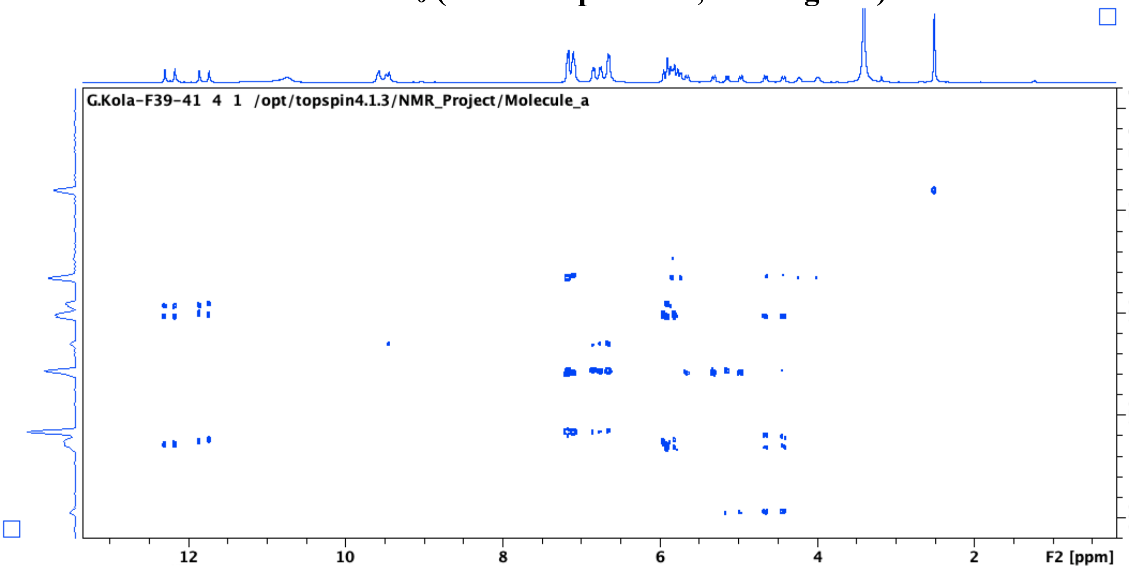

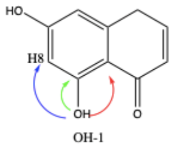

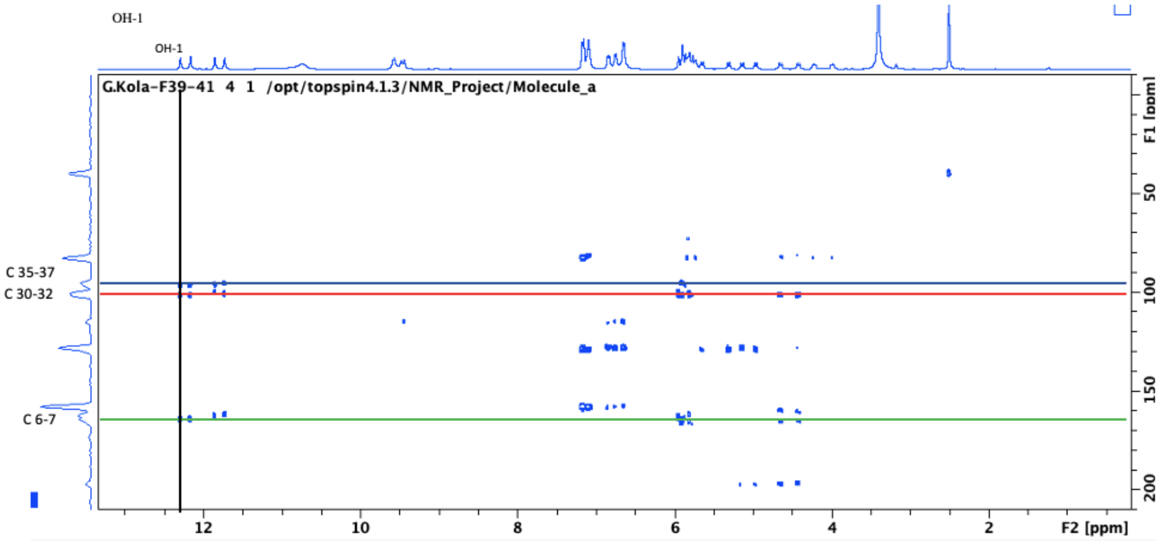

Supplement: Supplementary file 1 [file molecules-30-03813-s001.zip › molecules-3732239-supplementary.pdf]
